# Exploring Meaning-Making and Identity Transformation Among Adults Living with Type 2 Diabetes in Saudi Arabia: A Hermeneutic Phenomenological Study

**DOI:** 10.3390/healthcare14142208

**Published:** 2026-07-21

**Authors:** Nader E. Alotaibi, Omar Qaladi

**Affiliations:** 1 Department of Medical-Surgical Nursing, College of Nursing, King Saud University, Riyadh 12372, Saudi Arabia; 2 Department of Community and Psychiatric Mental Health Nursing, College of Nursing, King Saud University, Riyadh 12372, Saudi Arabia

**Keywords:** type 2 diabetes, lived experience, hermeneutic phenomenology, meaning-making, identity transformation, chronic illness, Saudi Arabia

## Abstract

Background: Diabetes mellitus is highly prevalent in Saudi Arabia and shapes individuals’ lived experiences within culturally and religiously embedded contexts. Although existing research has extensively examined biomedical, behavioral, and psychosocial dimensions of diabetes using quantitative and conventional qualitative approaches, there remains limited understanding of how individuals interpret, construct meaning, and reconstruct identity in relation to the illness over time. In particular, phenomenological inquiry is needed to access the lived, embodied, and existential dimensions of type 2 diabetes that are often not captured in existing outcome- or theme-focused qualitative studies. This study aimed to explore how Saudi adults with type 2 diabetes experience identity transitions, construct meaning, and undergo personal transformation. Methods: A hermeneutic phenomenological design informed by van Manen was employed. Twenty-seven Saudi adults with type 2 diabetes of at least one year’s duration were purposively recruited from a tertiary diabetes center in Riyadh. The sample size was guided by the principles of hermeneutic phenomenology, which prioritize depth, richness, and interpretive adequacy rather than numerical saturation. Recruitment and data collection continued until interpretive sufficiency was achieved, defined as the point at which no new experiential meanings or conceptual insights emerged across iterative analysis. Data were generated through in-depth, face-to-face interviews conducted in Arabic. Analysis followed an iterative interpretive process guided by the hermeneutic circle. Reflexivity, member checking, and peer review were used to enhance rigor. Results: Diabetes was experienced as a profound multidimensional disruption affecting embodiment, social roles, emotional well-being, and existential orientation. Participants described persistent fatigue and bodily limitation, shifting family roles and reduced social participation, emotional turbulence moving from shock to acceptance, and ongoing processes of meaning-making grounded in faith, gratitude, and strengthened family relationships. Conclusions: Living with diabetes involves an ongoing process of meaning-making and identity reconstruction that extends beyond clinical disease management to include emotional, social, and existential dimensions. These findings highlight the importance of culturally sensitive, person-centered care that addresses the lived experience of chronic illness within its sociocultural and spiritual context.

## 1. Introduction

Diabetes mellitus is one of the most prevalent and impactful chronic diseases of the 21st century, exerting a substantial burden on individuals, families, and healthcare systems worldwide [1]. Recent estimates indicate that 589 million adults are living with diabetes globally, with projections rising to 853 million by 2050 [2]. This increase is driven by factors such as population growth, aging, urbanization, and lifestyle changes [1,2]. In Saudi Arabia, the burden of diabetes is particularly high, with recent estimates indicating that more than 5.3 million adults are currently living with diabetes, and this number is projected to reach 9.5 million by 2050 [3].

In addition to epidemiological trends, understanding diabetes requires consideration of the ways people deal with and make sense of the condition in their everyday lives. While statistics show the prevalence of diabetes in the population and the problems related to its treatment and prevention, this information does not capture the embodied, emotional, and identity-related aspects of the condition—aspects that must be studied to understand the phenomenon from psychosocial, relational, and existential perspectives, thereby revealing the process of meaning-making within the context of chronic illness. Beyond its biomedical classification, type 2 diabetes mellitus (T2DM) is increasingly considered an illness that changes individuals’ everyday lives.

From a phenomenological perspective, chronic illness disrupts how individuals experience their body, themselves, and their daily life. Individuals are no longer able to take their body and health for granted and are forced to control and manage their body in terms of uncertainty and limitation [4]. People who have T2DM must face the issues associated with their diet, taking medications, blood glucose level regulation, and possible complications such as neuropathy, renal and heart diseases [5]. These challenges extend beyond physical discomfort to distress and disruption of social roles and daily life [5,6]. Living with diabetes therefore requires ongoing adaptation, through which individuals progressively reconstruct their sense of self. Importantly, these processes are not culturally neutral; they are shaped by the social and symbolic context in which they occur. In Saudi Arabia, this context is defined by strong family ties, collectivist values, and deeply rooted religious traditions [7,8]. Within this setting, illness is understood not only as a medical condition but as a moral, social, and spiritual experience—from an Islamic perspective, often interpreted as a test from God that offers meaning and an opportunity for spiritual growth. Family and societal expectations further shape how illness is perceived, managed, and disclosed [7,8,9]. Therefore, living with T2DM entails constant rebuilding of identity and meaning as influenced by the changing physical condition, social context, and spirituality. When there is inadequate cultural awareness, clinicians may provide health care services that are scientifically valid but culturally inappropriate.

Although there is a growing awareness about the psychosocial dimension of diabetes, the current literature is predominantly based on biomedical and behavioral methods of research, whereas little attention has been paid to the experiential and interpretative sides of the disease [10,11]. Some works examine emotional well-being and coping, but there is still limited insight into individual perceptions of the condition within cultural settings.

Phenomenology understands “the lived body” as the body experienced by individuals in their everyday life, rather than viewed from a medical standpoint, while “embodied experience” denotes the manner in which illness is perceived and incorporated through bodily awareness. In this regard, type 2 diabetes is described as a disruption of individuals’ habitual bodily and existential experience. Post-traumatic growth theory, which explains the processes of reconstruction and growth after a crisis, complements this framework.

Furthermore, despite the growing body of literature on the prospects for positive change (e.g., post-traumatic growth), such processes are rarely studied using culturally informed and phenomenologically oriented perspectives [12,13]. Therefore, it is crucial to address an existing gap in the literature: few culturally oriented studies have explored how Saudi adults with T2DM reconstruct their identities and lives. Filling this gap is important because it would help us better understand how people with chronic disease not only cope with illness but also experience their lives.

To address this need, the current study uses a hermeneutic phenomenological framework to investigate how Saudi adults living with type 2 diabetes perceive and understand their disease as a continuous process of identity construction and meaning-making within their specific culture. This approach is used to gain a deep contextual comprehension of the disease and to provide culturally appropriate care. Hermeneutic phenomenology was chosen as it allows for interpretive investigation of the way people make sense of type 2 diabetes in relation to the context in which they live. It differs from descriptive research methods in that it is based on interpretation and the collaborative construction of meaning between researcher and respondent.

## 2. Materials and Methods

### 2.1. Study Design

This study adopted a hermeneutic phenomenological approach informed by the philosophical writings of [14] and further articulated by Holloway and Galvin (2016) [15]. Van Manen’s hermeneutic phenomenology was utilized for its strong alignment with the study aim of exploring how individuals interpret and construct meaning around type 2 diabetes in their cultural and existential contexts. It is well suited to addressing lived experience, identity transformation, and meaning-making through an interpretive rather than purely descriptive focus. Its key principles, including the lifeworld existential, the hermeneutic circle, and reflective writing, guided iterative movement between participants’ accounts and developing interpretations throughout data collection and analysis [14,16]. This approach, grounded in interpretive phenomenology, enables in-depth engagement with participants’ narratives to uncover how meaning and personal transformation are experienced and interpreted over time. This approach aligns with an interpretivist epistemological stance, emphasizing the co-construction of meaning between researcher and participant. This interpretive stance informed all stages of analysis, where meanings were not treated as fixed but as emerging through ongoing interaction between participant accounts and researcher interpretation. In this study, diabetes was approached not merely as a clinical diagnosis, but as an experience that unfolds across bodily, emotional, social, and spiritual dimensions. Through this lens, participants’ accounts were understood as expressions of how living with type 2 diabetes mellitus (T2DM) becomes woven into personal identity, everyday practices, and broader life meanings within the Saudi cultural context. This study was reported in accordance with the Consolidated Criteria for Reporting Qualitative Research (COREQ) guidelines [17].

### 2.2. Participants and Sampling

The study included Saudi adults between 30 and 70 years of age who had been diagnosed with T2DM for at least one year. This duration allowed participants to reflect on the sustained impact of diabetes on their lives, identities, and perspectives over time. Eligibility criteria required participants to be Arabic-speaking, medically stable, and cognitively able to engage in in-depth interviews. Individuals who were terminally ill, diagnosed with severe psychiatric conditions, or cognitively impaired were excluded to ensure participant well-being and data quality.

Purposive sampling was used to recruit participants who could provide rich, reflective narratives. Attention was given to achieving variation in gender, age, educational background, socioeconomic status, and geographic location. This variation was essential to capturing variation in how meaning, identity, and illness experience are constructed across sociocultural contexts. Recruitment continued until thematic interpretive depth and phenomenological richness were achieved, whereby no new experiential meanings or interpretive insights emerged from successive interviews. A total of 27 participants were included, consistent with phenomenological research, where depth of understanding is prioritized over sample size.

Recruitment proceeded until interpretive sufficiency was attained, indicated by the absence of new experiential insights across consecutive interviews. This determination relied on iterative comparison of incoming data with developing interpretations during parallel data collection and analysis. Recruitment cessation was jointly determined by the principal researcher and a second qualitative researcher during regular analytic discussions. It was substantiated by iterative examination of interview transcripts, coding summaries, reflexive analytic notes, and systematically documented in a reflexive journal and audit trail to ensure transparency and auditability.

Participant recruitment was facilitated through collaboration with physicians and nursing staff working within the diabetes center. Clinical staff assisted in identifying individuals who met the eligibility criteria and provided a brief introduction to the study. To minimize potential selection bias, staff were instructed to approach a broad and diverse range of eligible participants rather than selectively recruiting based on specific characteristics. Where feasible, participants were approached consecutively to further reduce selection bias. Interested individuals were provided with an information sheet outlining the study purpose, procedures, and ethical considerations. They were encouraged to review the information carefully, discuss participation with family members if desired, and contact the researcher directly should they wish to participate.

A small number of eligible individuals declined participation (*n* = 6), due to time constraints and lack of interest. To reduce selection bias, recruitment was performed using consecutive sampling with staff instructed to approach a diverse range of eligible patients. Furthermore, recruitment was carefully monitored to ensure diversity in demographic and clinical characteristics, and participation remained entirely voluntary and free from clinical staff influence. Prior to each interview, the researcher reviewed the study details with participants, addressed any remaining questions, and obtained written informed consent. Participants also provided basic demographic information, including age, gender, educational level, place of residence, and duration of living with diabetes. This process was conducted in a manner that upheld voluntary participation, ensured informed decision-making, and respected cultural and personal considerations.

### 2.3. Study Setting

Data collection took place in a specialized diabetes center within a tertiary care hospital in Riyadh, Saudi Arabia. The center provides comprehensive, multidisciplinary care for adults with diabetes, including endocrinology services, nutritional counseling, and diabetes education, and serves a diverse patient population. Moreover, the center serves a large adult population with varied backgrounds, disease durations, and treatments, providing both routine and specialized diabetes care and supporting a heterogeneous sample relevant to the study aim.

Interviews were conducted in private rooms within the center to ensure confidentiality and participant comfort. Flexibility in interview location was offered, where feasible, to accommodate individual preferences and circumstances. This flexibility facilitated more open and reflective sharing of participants’ lived experiences.

### 2.4. Data Collection

This study used in-depth, semi-structured interviews lasting 60–90 min. Each interview was conducted face-to-face in Arabic by the principal researcher, who has formal training and experience in qualitative research and clinical nursing. The researcher shared linguistic and cultural familiarity with participants but had no prior relationship with them before recruitment. Every effort was made to maintain gender congruence throughout the study to account for cultural considerations. All interviews were audio-recorded with participants’ consent. Selected data were translated into English using a forward–backward translation approach to ensure semantic and conceptual equivalence. Probing questions and reflective prompts were used to deepen participants’ accounts and clarify meanings. Particular attention was given to preserving cultural meanings and contextual nuances during translation. In addition to interview data, field notes were recorded to capture non-verbal cues, contextual factors, and the researcher’s immediate reflections during and after interviews.

Table 1 presents the interview guide, titled “Meaning and Transformation”, which was developed based on a literature review of the phenomenology of chronic illness, spirituality, and identity transformation, and trialed with healthcare professionals in Saudi Arabia [18,19,20,21]. Semi-structured interviews explored experiences of life prior to and following diagnosis, emotional experiences, coping, changes in values, religious and cultural factors, and growth and transformation. A pilot testing of the interview was performed with two participants to assess clarity and relevance. Minor adjustments were made to question wording and sequencing to improve flow and depth, coherences, and alignment with the study objectives.

### 2.5. Data Analysis

The analysis employed a hermeneutic phenomenological approach [14], focusing on the interpretation of results. Analysis involved immersive and iterative reading of the transcripts to achieve deep familiarity with the data [22]. Analysis included identifying meaning units, and inductive coding key experiential statements. Codes were iteratively compared and organized into interpretive themes through cross-case analysis. Discrepancies were resolved collaboratively, with reference to transcripts and reflexive notes to ensure interpretive rigor.

Key phrases reflecting participants’ meaning-making and identity reconstruction were identified. Phenomenological meanings were interpreted through reflective engagement with participants’ narratives, focusing on how experiences were lived and understood rather than segmented into discrete analytical units. Interpretive themes were developed to illuminate essential structures of lived experience across participants’ accounts. Interpretation proceeded through continuous movement between parts and the whole, allowing deeper meanings to emerge through iterative engagement with the data. The analysis focused on uncovering underlying meanings, moving beyond description to interpret the lived and existential dimensions of participants’ experiences.

The analysis strictly followed the six steps of methodological activity proposed by van Manen: turning to the phenomenon of interest, exploration of the experience of living, reflection on essential themes, description through writing and revising, keeping a keen relation with the phenomenon, and the relation between parts and the whole [22]. NVivo 14 (Lumivero, Denver, CO, USA) was used as an organizational tool to manage the data, while interpretation remained grounded in reflective and iterative engagement with participants’ narratives [23]. Reflexive journaling supported ongoing critical self-awareness and enhanced interpretive rigor. The second qualitative researcher also reviewed transcripts and emerging interpretations.

### 2.6. Researcher Reflexivity

Reflexivity was embedded throughout data collection and analysis. The principal researchers regularly recorded assumptions, emotional responses, and interpretive decisions immediately after each interview. These reflections were then reviewed with the second researcher during scheduled analytic meetings, to explore alternative interpretations and reduce bias. The influence of clinical background and shared cultural context on data collection and interpretation was also considered.

The principal researcher is a registered nurse with clinical experience in diabetes care and training in hermeneutic phenomenology, which assists with clinically and culturally informed engagement with participants’ narratives. The second researcher is a qualitative scholar with expertise in interpretive methodologies who contributed independent analytical review throughout the study.

No prior therapeutic or personal relationships existed between the researchers and participants. However, the researchers’ familiarity with the language and culture in the Saudi context facilitated rapport while requiring reflexive attention to interpretive assumptions.

Ongoing reflective journaling and peer debriefing assisted with maintaining reflexivity to ensure clinical and cultural perspectives shaped, rather than biased, interpretation of the findings.

### 2.7. Trustworthiness

Trustworthiness was established using Lincoln and Guba’s model of 1985 [24]. Credibility was enhanced through prolonged engagement with the data, iterative analysis, and member checking to ensure alignment between participants’ accounts and their interpreted meanings. Transferability was supported by the provision of rich, contextualized descriptions of participants’ sociocultural environments. Dependability was ensured through an audit trail documenting methodological decisions and analytical processes. Confirmability was strengthened through reflexive journaling, peer debriefing, and systematic comparison between data and emerging interpretations. In addition, data triangulation was achieved through the integration of interview transcripts, field notes, and reflexive records, allowing for a more comprehensive and nuanced understanding of participants’ experiences. Rigor was further strengthened through sustained interpretive engagement with the data throughout the analytic process.

### 2.8. Ethical Considerations

Ethical approval for this study was obtained from the King Saud University Institutional Review Board (IRB) prior to the commencement of data collection (Approval No. E-25-10062; 1 January 2026). Informed written consent was obtained from participants, who were informed of the voluntary nature of the study, the protection of their confidentiality, and their right to withdraw without repercussions. Pseudonyms were used instead of other identifiers, and the data was stored on encrypted devices and in locked cabinets. Participants were free to withdraw or pause the interview at any time, and they were provided with referrals for counseling, if needed. No incentives that could coerce them were used.

## 3. Results

### 3.1. Participant Characteristics

As shown in Table 2, the study included 27 adults living with type 2 diabetes mellitus. Participants were predominantly middle-aged, with a mean age of 52.6 years (range 32–69), and had been living with diabetes for an average of 9.1 years (range 1–25). The sample included a nearly equal distribution of men and women and reflected variation in educational attainment, ranging from no formal education to university-level qualifications. Most participants were married, and over half were not currently employed or were retired.

Clinically, participants presented with diverse treatment regimens, including oral medications, insulin therapy, or a combination of both. The majority had suboptimal glycemic control, as reflected in elevated HbA1c levels, and many reported living with at least one diabetes-related complication, such as neuropathy or retinopathy. Overweight and obesity were common within the sample.

### 3.2. Themes Derived from the Analysis

**Figure 1:**
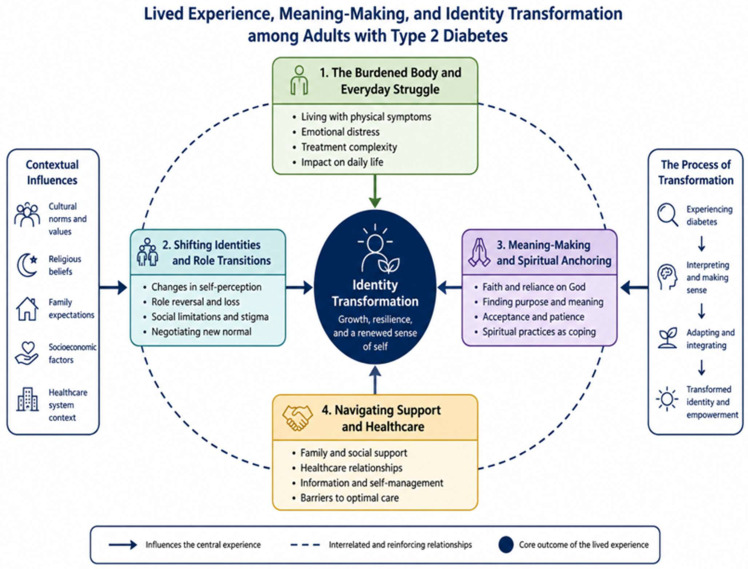
The relationships among themes and subthemes.

The analysis revealed four interrelated themes that illuminate how diabetes is experienced as a multidimensional disruption that recon rRes embodiment, identity, social positioning, and existential orientation. These themes reflect the dynamic process through which individuals interpret, negotiate, and reconstruct their lived experience over time. An overview of these themes is presented in Table 3. The relationships among themes and subthemes are illustrated in Figure 1.

The experience of embodied disruption was reported consistently across the majority of participants, regardless of age, gender, or duration of diabetes. While all participants described some degree of bodily change associated with diabetes, the intensity and specific emphasis of individual elements (e.g., fatigue, pain, or fear of complications) varied across participants.

### 3.3. Theme 1. Embodied Disruption: Living with a Changed Body

#### 3.3.1. Subtheme 1.1: Fatigue, Weakness, and Altered Energy

Participants repeatedly described a pervasive sense of fatigue that shaped their daily functioning. Many emphasized its persistence and the way it disrupted routine activities and energy levels.

“*I always feel so tired… I sleep tired, and I wake up tired*” (P4).

“*Sometimes I cannot even hold myself when I stand up, I feel dizzy and drained*” (P12).

“*Before I got diabetes… I get tired so easily now*” (P3).

Fatigue was commonly expressed as continuous and difficult to overcome, affecting participants’ ability to sustain previous levels of activity.

#### 3.3.2. Subtheme 1.2: Pain, Discomfort, and the Unpredictable Body

Participants described physical discomfort associated with fluctuations in blood glucose levels, often characterizing these episodes as distressing and unpredictable.

“*When my blood sugar goes up to 300 or 400… my whole body hurts*” (P14).

Several accounts emphasized the uncertainty of symptoms and their sudden onset, which made bodily experiences feel less stable and harder to anticipate.

#### 3.3.3. Subtheme 1.3: Everyday Restrictions and Food-Related Losses

Food was frequently described as a source of restriction and emotional loss, particularly in social and family contexts.

“*I do not really enjoy family gatherings anymore… I cannot eat what I want*” (P6).

“*Whenever I see knafeh… my chest feels tight*” (P11).

“*My older sons… keep watching what I am eating*” (P22).

Participants described changes in eating practices that affected both everyday enjoyment and participation in shared family meals.

#### 3.3.4. Subtheme 1.4: Fear of Future Complications

Many participants expressed concerns about future health deterioration, often shaped by observing complications in others or imagining possible outcomes.

“*When I see someone lose a leg… I feel like it is going to be my turn*” (P8).

“*Sometimes I picture myself with one leg gone… it really makes me scared*” (P26).

“*I am always on edge… worrying about what could happen*” (P15).

These accounts reflect ongoing concern about disease progression and its potential impact on future bodily functioning.

Across accounts, participants described diabetes as involving changes in bodily functioning, energy, and everyday physical experience. These changes were reflected in persistent fatigue, episodic discomfort, altered eating practices, and concerns about future health. Collectively, these experiences illustrate how living with diabetes is encountered through ongoing bodily awareness and adjustment in daily life.

### 3.4. Theme 2. Loss, Burden, and Shifting Social Identity

#### 3.4.1. Subtheme 2.1: Dependency and Role Reversal

Participants described changes in family roles and increasing reliance on others in daily life. Many contrasted their previous independence with their current need for assistance.

“*I used to take care of my kids… now it is the opposite*” (P7).

“*Even when I need to take insulin, I need my son or my wife to help*” (P20).

“*I was the strong one… now my wife looks at me with worry*” (P23).

“*My kids do all the shopping now*” (P19).

These accounts reflect changes in everyday functioning within family life, particularly in relation to self-care and household responsibilities.

#### 3.4.2. Subtheme 2.2: Social Withdrawal and Reduced Participation

Participants reported reduced engagement in social and community activities, often linked to practical concerns or discomfort in managing their condition outside the home.

“*I stopped attending many weddings… I have been going out less*” (P14).

“*Even during Eid, I stay home*” (P16).

“*I worry I will forget my medicine… so I decide not to go*” (P12).

“*Sometimes I hold back because I feel like I need to pee*” (P18).

These experiences show how participation in social events became more limited over time for some participants.

#### 3.4.3. Subtheme 2.3: Worry About Burdening Family and Future Dependence

Many participants expressed concern about becoming dependent on family members in the future and the potential impact of their illness on household responsibilities.

“*I worry my kids might have to leave their jobs to take care of me*” (P8).

Such concerns influenced how participants thought about their illness and their future needs, particularly in relation to family support.

### 3.5. Theme 3. Emotional Turbulence to Acceptance: The Inner Journey of Adaptation

#### 3.5.1. Subtheme 3.1: Shock, Denial, and Resistance

Participants described diagnosis as an unexpected and distressing moment, often accompanied by difficulty accepting its permanence.

“*I could not believe it… it hit me like a punch*” (P1).

“*I went to several doctors… hoping it was temporary*” (P27).

“*It felt like a nightmare… but it turned out real*” (P5).

These accounts reflect initial reactions marked by disbelief and attempts to reinterpret or challenge the diagnosis.

#### 3.5.2. Subtheme 3.2: Anxiety, Fear, and Loss of Control

Many participants described ongoing worries related to managing diet, symptoms, and blood sugar fluctuations.

“*I am scared to eat, scared to drink… I cannot live normally*” (P2).

“*I started checking my sugar many times a day… it made me anxious*” (P13).

“*My life is not the same… nothing feels fun anymore*” (P5).

“*Life just wears me down sometimes*” (P15).

These experiences show how day-to-day management of diabetes became closely tied to emotional strain and persistent worry for many participants.

#### 3.5.3. Subtheme 3.3: Gradual Acceptance and Redefining Normality

Some participants described adjusting over time by incorporating diabetes management into daily routines and lifestyle choices.

“*I realized this illness is not going away… I just wanna take my meds and deal with it.*” (P9).

“*The best thing is to exercise and improve my lifestyle… my habits will stay with me.*” (P10).

These accounts reflect ways in which participants described adjusting their routines and expectations in response to living with a long-term condition.

Across accounts, participants described varied emotional responses to diagnosis and ongoing management, including initial shock, sustained worry, and gradual adjustment. These experiences illustrate how emotional responses to diabetes evolve over time as individuals navigate uncertainty and adjust to long-term illness in everyday life.

### 3.6. Theme 4. Meaning-Making and Personal Transformation Through Faith, Values, and Relationships

#### 3.6.1. Subtheme 4.1: Faith as a Framework for Understanding Illness

Participants described faith as an important way of understanding and coping with their experience of diabetes. Many interpreted their condition within religious meanings.

“*God gave me this illness to teach me patience… it is not a punishment.*” (P17).

“*Diabetes brought me closer to Allah… I learned to trust Him.*” (P21).

These accounts show how religious belief was used to make sense of illness and its challenges.

#### 3.6.2. Subtheme 4.2: Gratitude, Empathy, and Value Reorientation

Some participants described changes in how they viewed daily life and personal priorities.

“*Blessings are not about money… I say alhamdulillah… I try to enjoy every bit of life.*” (P24).

“*Before, I never really got what others were going through… now I feel their pain*” (P25).

These experiences reflect changes in how participants spoke about appreciation, awareness of others, and everyday priorities.

#### 3.6.3. Subtheme 4.3: Strengthened Family Bonds and Reprioritization

Participants also described changes in family relationships and the importance of support over time.

“*My family got closer… we check on each other… we are a team*” (P11).

Many noted that family relationships became more central in their daily lives and decision making.

Participants described understanding and responding to diabetes through faith, evolving values, and strengthened family relationships, reflecting shifts in perspectives and priorities. Family relationships became increasingly significant in daily life and decision-making. However, meaning-making and positive transformation were not universal, being more evident among those with longer disease duration and stronger family or religious support. In contrast, recently diagnosed participants or those with complications or limited support more often reported fear, anxiety, and distress. Overall, identity transformation was variable, shaped by illness duration, clinical burden, and relational and spiritual resources.

## 4. Discussion

This study contributes to the existing literature by framing chronic illness phenomenologically as an existential and relational process of becoming, rather than merely behavioral adaptation. It extends existing frameworks within a culturally embedded context, highlighting lived experience as a dynamic site of identity and meaning reconstruction.

The findings extend beyond description to show how individuals actively interpret and reconstruct their experiences under ongoing uncertainty. Accounts reported not only persistent symptoms but also disruption to body, identity, and sense of continuity over time, prompting ongoing meaning-making as individuals navigate changing abilities, roles, and emotional complexities. From a phenomenological lens, this reflects both physical suffering and an existential shift in the relationship between body, self, and world.

Adaptation can be understood as a dynamic and non-linear process shaped by personal, cultural, and spiritual resources. This highlights the need for healthcare approaches that move beyond symptom management to underpin patients’ identity reconstruction, emotional adjustment, and meaning-making within their sociocultural contexts.

While the disruption of embodiment, identity, and meaning is well established in phenomenological and qualitative health research, the present study contributes by illustrating how these processes are specifically shaped within a Saudi, family-oriented, and Islamic cultural context.

The four-theme trajectory can be situated within three frameworks. It confirms Bury’s [25] biographical disruption model, with movement from embodied disruption and role loss to emotional adjustment and reconstructed meaning. It extends this by showing biographical repair is primarily achieved through collective and spiritual resources (family and faith) rather than individual cognitive reappraisal [26,27]. It partially aligns with post-traumatic growth theory [28], but challenges its individualistic framing by emphasizing socially and religiously co-constructed growth [28]. The oscillation in Theme 3 further supports dialectical “biographical flow” models over linear stage approaches [29].

In the first theme, diabetes emerges as a profound disruption of the lived body, characterized by weakness, pain, dietary disruption, and fear of impending complications. These disruptions align with the conceptualization of the phenomenological effects of chronic illness, depicted as an “existential assault” on the body [30]. The findings regarding perpetual exhaustion and decreased physical strength extend previous research by demonstrating that fatigue is experienced as one of the most debilitating aspects of diabetes among populations in the Saudi Peninsula [11,20].

Accounts of persistent fatigue reveal how exhaustion is experienced not merely as a symptom, but as a disruption to agency and continuity in everyday life. Positioned within the existing literature, fatigue emerges as a significant barrier to self-care and mobility [31], while the present findings extend this understanding by demonstrating how exhaustion fundamentally alters individuals’ capacity to sustain engagement in daily life [32,33,34,35]. This disruption repositions the body from a taken-for-granted medium of action to an object of constant awareness and limitation.

Pain associated with hyperglycemia provides further insight into how fluctuations in blood glucose reshape bodily perception and control, suggesting that variability in blood sugar undermines bodily confidence and results in the development of a state of hypervigilance [36,37,38]. As a chronic stressor characterized by unpredictable glycemic volatility, diabetes mellitus often engenders a heightened perceived symptom burden alongside a diminished sense of personal control [39]. This psychological vulnerability fosters acute health anxiety, precipitating a state of somatic hypervigilance. Consequently, individuals engage in continuous bodily scanning, misinterpreting benign physiological sensations as indicative of catastrophic physical regression and disease progression.

Food restrictions were strongly linked with cultural identity and rituals. Consistent with the findings of studies among the Arabic population, where the participants were sad about the loss of local desserts and meals, as the primary function of meals is hospitality and belonging [40,41]. The discomfort of family monitoring of diet is also consistent with previous findings that diet monitoring is a source of family tension and loss of autonomy [42].

Fear of amputation, blindness, and disability is well-documented in the literature of Saudi Arabia and the Middle East [43,44,45]. In the Middle East and Saudi Arabia, the psychological burden of diabetes is acutely driven by fears of microvascular and macrovascular complications, specifically lower-limb amputation, loss of vision, and permanent disability [46,47]. These regional anxieties are continuously reinforced by high epidemiological rates of poorly controlled glycemic levels, which directly correlate with elevated local incidences of diabetic retinopathy and advanced foot ulcers [44]. Ultimately, the chronic anticipation of these catastrophic outcomes functions as a localized stressor that severely undermines the psychological well-being and treatment adherence of patients across the Arab population [43].

However, comparison with international qualitative studies suggests that embodied disruption is not experienced uniformly across contexts. For example, a Belgian study of individuals with advanced T2DM framed bodily deterioration largely in terms of fragmented health-system navigation rather than cultural threat [48], while a Nepalese study emphasized the practical impact of bodily limitations on daily self-care with minimal spiritual interpretation [49]. These contrasts indicate that although symptoms such as fatigue, pain, and fear of complications may be widely shared, their meaning varies across settings. Embodied experiences may be understood as existential, structural, or practical, depending on cultural and health-system contexts, rather than reflecting a single universal phenomenological pattern of chronic illness.

Overall, the discussed theme demonstrates how diabetes affects bodily independence, reconfiguring the meaning of the physical self, which resonates with phenomenological conceptualizations of the “broken lived body”. Taken together, these findings extend the existing literature by demonstrating that bodily disruption is not merely functional, but fundamentally alters how individuals inhabit and relate to their bodies over time.

The second theme demonstrates how diabetes reconfigures family dynamics, leading to greater reliance on others and withdrawal from social engagements. These findings demonstrate how chronic illness is not merely experienced at an individual level, but is deeply embedded within culturally structured social roles that shape identity, responsibility, and relational expectations.

In the first subtheme, many participants reported transitioning from the caregiving role to the cared-for role, a transition previously identified in qualitative research of Italian patients with chronic diseases [50,51]. Role reversal is particularly difficult in male- and family-oriented societies, where autonomy and a sense of being a provider are associated with male esteem [52,53]. In this context, the disruption of provider roles appears to challenge not only functional independence but culturally embedded expectations of masculinity and responsibility. The loss of male authority among participants aligns with the literature indicating that chronic illness reshapes gender roles within Saudi households [52,53].

Fear of symptoms, dietary restrictions, and embarrassment prevented patients from attending wedding ceremonies, Eid celebrations, and other social events [54]. In Middle Eastern and South Asian contexts, cultural celebrations like Eid and weddings center on high-glycemic communal meals, creating a profound psychosocial dilemma for individuals with diabetes. Declining food violates hospitality norms and risks stigmatizing disclosure, while indulging threatens acute glycemic complications. Consequently, patients frequently utilize social isolation as a defensive coping mechanism to avoid both cultural friction and metabolic instability, despite its significant psychological toll [54,55,56].

Social avoidance is worse for patients when cultural events revolve around food, thus increasing the social costs of avoiding these events [54,56]. The idea of forcing family members to provide caregiving support is also informed by findings that the fear of dependence is among the factors that contribute significantly to the level of emotional distress among patients with DM [57].

In these mutual societies, illness is viewed relationally, and the fear of dependence, thus the burden, surpasses the illness itself [57]. A more critical reading against international evidence, however, complicates the assumption that role reversal and social withdrawal are primarily products of collectivist family structures. An intersectional qualitative study of adults with diabetes in Canada found that disrupted identity and withdrawal from social participation were driven less by family-role expectations than by experiences of stigma, discrimination, and microaggressions linked to gender, race, and age, with resilience—rather than dependency—emerging as the organizing concept [58].

The third theme reveals that participants’ emotional experiences unfolded as a dynamic and non-linear process: shock and denial, fear and hopelessness, culminating in acceptance and adaptation. Denial of diagnosis reflects the profound existential disruption associated with chronic illness onset, where individuals struggle to reconcile the permanence of the condition with prior assumptions of health, as reported by Patierno and his colleagues (2023) in their systematic review [57,59,60]. Obtaining multiple opinions to challenge the diagnosis is also consistent with findings from Kuwait and Bahrain studies [59,61].

Fear of food, surveillance routines, and comorbidities confirms the documented psychological concerns of Saudis with diabetes, who are known to experience increased levels of anxiety and depressive symptoms [20]. Accounts of living “wearing them down” reflect the international literature that finds excessive fear of unpredictable glycemic events reduces their emotional well-being [38,62].

Acceptance occurred via lifestyle modifications, consistent with findings of diabetes behavioral studies, where the acknowledgment of the persisting nature of the illness, self-efficacy, and support of the family help adjustment [18,20]. Changes among the participants mirror the “reconstruction of normalcy” concept elaborated by the phenomenology of chronic illness studies, where living is routinized around the new physicalness [63,64]. In summary, this theme highlights the psychological work of living with diabetes, recognizing that emotional adjustment is a critical aspect of coping.

The fourth thematic heading encompasses the deep existential and spiritual work that participants undertake to incorporate diabetes into their lives, and it aligns well with the study’s objective of exploring meaning. This finding can be interpreted through a meaning-making lens, where religious belief systems function as interpretive frameworks that transform suffering into a morally and spiritually coherent experience. Often, the disease was seen as an exam by God, a view well documented in the qualitative literature of Saudi Arabia and consistent with Islamic beliefs about endurance, hardship, and the wisdom of God [65,66].

This finding underscores the central role of spirituality in how individuals make sense of living with diabetes. Consistent with the Integrative Process Model, participants engaged in an ongoing search for meaning in the face of chronic uncertainty, often interpreting their illness through a religious lens. Framing diabetes as a test or trial from God allowed participants to render suffering morally coherent and spiritually meaningful, aligning with broader belief systems that emphasize patience, endurance, and trust in divine wisdom. This highlights how spiritual and faith-based interpretations are not peripheral but integral to the process of adaptation, shaping how individuals understand, accept, and live with the condition over time [65,66]. This view of illness was also observed among Muslim populations with chronic illness in Iran, Pakistan, and Saudi Arabia, where religious beliefs promote spiritual growth [65,67,68,69,70].

The awakening of gratitude and empathy supports the literature on chronic illness, suggesting that the experience of adversity can serve as a trigger for constructive psychological growth [71]. Correspondingly, the literature on diabetes in the Saudi context also suggests that diabetes triggers a reappraisal of values and a gratitude for good health and relationships [72]. This reappraisal of values suggests that meaning-making offers a mechanism for transformation not only for coping purposes but also for post-illness transformation.

Family cohesion emerged as a prominent dimension of participants’ experiences, aligning with Saudi cultural practices that promote collective support and parental responsibility [10,52]. Increased family cohesion has also been observed among families living with diabetes worldwide [73,74]. This shift in relationship dynamics aligns well with the existing theorization of chronic illness, suggesting that the threat of disease triggers a relational closeness and new identity for patients [74]. Collectively, these findings suggest that diabetes functions not only as a source of disruption but as a catalyst for existential and relational reorientation, through which individuals actively reconstruct meaning, identity, and connectedness.

Across themes, the findings suggest that living with diabetes is best understood as a dynamic process in which bodily disruption, social identity, emotional adaptation, and meaning-making are not separate dimensions but mutually constitutive dimensions of experience. This integrative perspective advances phenomenological understandings of chronic illness by highlighting how individuals continuously negotiate their relationship with self, body, and world over time. These findings underscore the imperative to integrate psychosocial and meaning-centered approaches into diabetes care, particularly in culturally embedded contexts such as Saudi Arabia, where illness is experienced not only biologically but also relationally and spiritually. Importantly, these findings highlight the critical role of nurses in delivering culturally sensitive, person-centered care that addresses not only clinical management but also the emotional, social, and existential dimensions of chronic illness.

Taken together, the study refines existing phenomenological understandings by demonstrating how lived experiences of diabetes are culturally embedded, rather than proposing entirely new conceptual categories.

### 4.1. Implications

The implications of this study are best understood in relation to participants’ lived experiences of diabetes as an embodied, relational, and existential condition. The findings demonstrate that diabetes extends beyond biomedical management to involve disruptions to the body, shifts in identity, and ongoing efforts to make sense of these changes. Participants’ accounts highlight how physical alterations and fears of deterioration are closely tied to changing self-perceptions, underscoring diabetes as both a bodily and identity-transforming experience.

Within this interpretive context, faith emerged as a central resource in meaning-making, with many participants framing diabetes as a test from God. This spiritual orientation enabled individuals to situate their experiences within a broader moral and existential narrative emphasizing patience, acceptance, and trust in divine wisdom, suggesting the importance of sensitivity to spiritual beliefs in understanding how chronic illness is lived in culturally grounded contexts. Similarly, family relationships played a central role in shaping both support and tension in daily diabetes management. While family involvement provided emotional and practical assistance, it was also associated with concerns about autonomy, surveillance, and perceived burden, highlighting the need to attend to relational dynamics within care encounters.

Because the present findings indicate that positive adaptation was unevenly distributed across participants—appearing more consistently among those with longer disease duration, fewer complications, and stronger family/religious support—care providers should avoid treating acceptance or spiritual growth as an expected or required outcome. Newly diagnosed patients, those with significant complications, or those lacking strong family support may instead require sustained emotional and practical support rather than encouragement toward premature acceptance.

Taken together, these findings suggest that care approaches should be attentive to spirituality, family context, and the lived experience of embodied and identity-related change. However, these implications are interpretive and context-specific, and they do not indicate the effectiveness of any intervention. Rather, they reflect how individuals make sense of and negotiate chronic illness within their cultural and relational worlds, and they are intended to inform reflection on care practices rather than to establish or evaluate outcomes.

### 4.2. Limitations

This study should be interpreted in light of several limitations. First, participants were recruited from a single tertiary diabetes center in Riyadh, which may limit the transferability of findings to individuals receiving care in primary or secondary healthcare settings, as well as to those living in rural or remote areas where access to specialist services and structured follow-up may differ.

Second, the sample included medically stable adults with type 2 diabetes who were able to participate in in-depth interviews. As a result, the findings may not fully reflect the experiences of individuals with advanced complications, acute illness, or significant physical, cognitive, or health literacy limitations, who may articulate and interpret their experiences differently.

Third, although a forward–backward translation process was used to enhance semantic and conceptual equivalence, some loss of culturally embedded meaning may have occurred during translation from Arabic to English, particularly in relation to religious expressions, idiomatic language, and culturally specific understandings of illness.

Fourth, the cultural and religious context may have contributed to socially and religiously desirable responses, with participants potentially emphasizing narratives of acceptance, faith, or resilience. In addition, the semi-structured interview format may have encouraged reflection on change and adaptation. Although reflexivity, rapport building, and open-ended questioning were used to minimize these influences, such factors remain inherent in qualitative research and should be considered in interpretation.

Finally, consistent with interpretive phenomenology, the findings represent a co-constructed and contextually situated interpretation rather than an objective or generalizable account.

## 5. Conclusions

This phenomenological study provides an in-depth understanding of the lived experience of diabetes mellitus in Saudi Arabia. The findings indicate that living with diabetes is a life-disrupting experience that reconfigures one’s body, identity, relationships, emotions, and meaning. Despite these challenges, some participants described pathways of acceptance, strengthened faith, reshaped values, and enhanced family bonds. These findings suggest that diabetes is not only a chronic medical condition but also an ongoing existential process shaped by cultural, religious, and familial contexts. Importantly, these insights highlight the need for culturally sensitive, person-centered approaches in healthcare that address not only clinical management but also the emotional, social, and existential dimensions of living with diabetes. Future longitudinal and cross-cultural comparative research is needed to determine how these processes evolve over time and whether they generalize beyond the Saudi context.

## Figures and Tables

**Table 1:** Semi-structured interview guide.

Thematic Area	Interview Questions/Focus Areas
Opening and context	Life before diagnosis; experience of diagnosis; changes in life since diagnosis.
Understanding of diabetes	Meaning of diabetes; change in understanding over time; explaining condition to others.
Emotional experience and meaning	Emotions living with diabetes; difficult/meaningful experiences; impact on life priorities and sense of ‘good life’; gratitude and appreciation changes.
Identity, relationships and cultural/religious context	Changes in self-identity; effects on family/social roles; cultural expectations; coping beliefs and spirituality; diabetes as test/challenge/blessing.
Future outlook and reflection	Motivation for management; future expectations; advice to newly diagnosed; overall reflection on lived experience.

**Table 2:** Demographic and Clinical Characteristics of Participants (*N* = 27).

Characteristic	Category	*n* (%) or Mean ± SD (Range)
**Age (years)**	Mean ± SD	52.6 ± 10.4 (32–69)
	30–39	4 (14.8%)
	40–49	6 (22.2%)
	50–59	9 (33.3%)
	≥60	8 (29.6%)
**Gender**	Male	14 (51.9%)
	Female	13 (48.1%)
**Duration of Diabetes (years)**	Mean ± SD	9.1 ± 6.3 (1–25)
	1–5 years	8 (29.6%)
	6–10 years	7 (25.9%)
	11–15 years	6 (22.2%)
	>15 years	6 (22.2%)
**Educational Level**	No formal education	3 (11.1%)
	Primary education	5 (18.5%)
	Secondary education	8 (29.6%)
	University degree or higher	11 (40.7%)
**Employment Status**	Employed	12 (44.4%)
	Unemployed/Retired	15 (55.6%)
**Marital Status**	Married	23 (85.2%)
	Single/Widowed/Divorced	4 (14.8%)
**Treatment Type**	Oral hypoglycemic agents only	11 (40.7%)
	Insulin therapy	7 (25.9%)
	Combination (oral + insulin)	9 (33.3%)
**Presence of Diabetes-related Complications**	None reported	10 (37.0%)
	At least one complication	17 (63.0%)
**Type of Complications** *	Neuropathy	8 (29.6%)
	Retinopathy	6 (22.2%)
	Cardiovascular conditions	5 (18.5%)
	Diabetic foot issues	3 (11.1%)
**Body Mass Index (BMI)**	Mean ± SD	30.8 ± 5.2
	Overweight (25–29.9)	9 (33.3%)
	Obese (≥30)	15 (55.6%)
**Glycemic Control (HbA1c)**	Mean ± SD	8.4 ± 1.6%
	Controlled (<7%)	6 (22.2%)
	Uncontrolled (≥7%)	21 (77.8%)

* Participants may have reported more than one type of complication; therefore, frequencies may exceed the number of participants with complications.

**Table 3:** Themes and subthemes derived from participants’ narratives.

Theme	Subthemes	Analytical Description
Embodied Disruption: Living with a Changed Body	1.1 Fatigue and weakness 1.2 Pain and unpredictable symptoms 1.3 Food restrictions and loss of pleasure 1.4 Fear of complications	Diabetes was experienced as bodily disruption characterized by persistent fatigue, symptomatic unpredictability, dietary constraint, and anticipatory fear of future deterioration.
Loss, Burden, and Shifting Social Identity	2.1 Dependency and role reversal 2.2 Social withdrawal 2.3 Fear of burden and future dependence	Participants described diminished autonomy, altered social participation, and anxiety about becoming dependent on family members.
Emotional Turbulence to Acceptance	3.1 Shock and denial 3.2 Anxiety, fear, hopelessness 3.3 Acceptance and redefining normality	The emotional trajectory moved from initial disruption (shock and fear) toward gradual psychological adjustment and redefinition of everyday life.
Meaning-Making and Personal Transformation	4.1 Faith and spiritual meaning 4.2 Gratitude and value transformation 4.3 Family closeness and reprioritization	Participants engaged in meaning-making through spirituality, value reorientation, and strengthened relational bonds.

## Data Availability

The datasets generated and/or analyzed during the current study are available from the corresponding author upon reasonable request. The data are not publicly available due to privacy and ethical restrictions.
